# A systematic review of the pharmacological modulation of autobiographical memory specificity

**DOI:** 10.3389/fpsyg.2022.1045217

**Published:** 2022-11-14

**Authors:** Emma Cawley, Giulia Piazza, Ravi K. Das, Sunjeev K. Kamboj

**Affiliations:** Research Department of Clinical, Educational and Health Psychology, University College London, London, United Kingdom

**Keywords:** autobiographical, memory, specificity, pharmacological, modulation

## Abstract

**Background:**

Over-general autobiographical memory (AM) retrieval is proposed to have a causal role in the maintenance of psychological disorders like depression and PTSD. As such, the identification of drugs that modulate AM specificity may open up new avenues of research on pharmacological modeling and treatment of psychological disorders.

**Aim:**

The current review summarizes randomized, placebo-controlled studies of acute pharmacological modulation of AM specificity.

**Method:**

A systematic search was conducted of studies that examined the acute effects of pharmacological interventions on AM specificity in human volunteers (healthy and clinical participants) measured using the Autobiographical Memory Test.

**Results:**

Seventeen studies were identified (986 total participants), of which 16 were judged to have low risk of bias. The presence and direction of effects varied across drugs and diagnostic status of participants (clinical vs. healthy volunteers). The most commonly studied drug—hydrocortisone—produced an overall *impairment* in AM specificity in healthy volunteers [*g* = −0.28, CI (−0.53, −0.03), *p* = 0.03], although *improvements* were reported in two studies of clinical participants. In general, studies of monoamine modulators reported no effect on specificity.

**Conclusion:**

Pharmacological enhancement of AM specificity is inconsistent, although monaminergic modulators show little promise in this regard. Drugs that reduce AM specificity in healthy volunteers may be useful experimental-pharmacological tools that mimic an important transdiagnostic impairment in psychological disorders.

**Systematic review registration:**

PROSPERO, identifier CRD42020199076, https://www.crd.york.ac.uk/prospero/display_record.php?ID=CRD42020199076.

## Introduction

Autobiographical memory (AM) refers to the store of self-related knowledge and memories for personal experiences (Conway and Pleydell-Pearce, 2000; Tulving, 2002). It is central to an individual's sense of self, identity, and their capacity to understand their place in the world. Dysfunctions in AM processing may therefore have serious implications for mental wellbeing. Much of the interest in AM has focused on the (deficits in) retrieval of *specific* AMs (i.e., spatiotemporally unique memories of personally experienced events). Although particularly well-characterized in depressive (Williams et al., 2007) and traumatic stress disorders (Moore and Zoellner, 2007; Ono et al., 2016), reduced retrieval of specific AMs, otherwise known as *overgeneral* AM, is found in a range of psychological disorders (e.g., Jones et al., 1999; Berna et al., 2016; Barry et al., 2021). In sufferers of depression, for example, maladaptive processing of memories for personal experiences and self-related knowledge may include overgeneral AM, reduced recollection of positive AM, and enhanced (repetitive and involuntary) access to negative self-representations and AMs (Dalgleish and Werner-Seidler, 2014). While these particular biases represent distinct phenomena, they all rely on dysfunctional or biased memory *retrieval* processes (Brewin, 2006), which are likely further compounded by negative affective biases (e.g., Hitchcock et al., 2020) and impaired or overgeneralized encoding of experiences (Dillon and Pizzagalli, 2018; Kumar et al., 2018; Murphy et al., 2019; Ai et al., 2020).

Pharmacological agents have been used to model neuropsychological dysfunctions in *encoding* or consolidation stages of memory processing (e.g., Kamboj and Curran, 2006). Such approaches have been invaluable in the study of organic brain disorders like Alzheimer's disease through the ability to replicate the commonly observed memory encoding/consolidation impairments that characterize these disorders (e.g., Haider et al., 2016). *Psychological* disorders, however, are characterized by more complex patterns of hyper- and/or hypomnesia that reflect dysfunctional memory storage and retrieval, yet evidence suggests that acute impairments to episodic memory *retrieval* can also be induced pharmacologically (e.g., Strange et al., 2003; Kuhlmann et al., 2005; Hurlemann et al., 2007; Diekelmann et al., 2011; Rimmele et al., 2015; Kroes et al., 2016). Retrieval dysfunction has largely been induced by agents targeting stress-related hormones and neurotransmitters [i.e., endogenous regulators of glucocorticoids and (nor)adrenaline]. These findings highlight the potential for developing pharmacological models of and, by extension, potential therapeutic targets for dysfunctional retrieval. However, unlike episodic encoding impairments in Alzheimer's, for example, there are currently no accepted experimental-pharmacological models of the transdiagnostic dysfunctional retrieval processes observed in common psychological disorders like depression, anxiety, and PTSD.

### AM specificity and psychological disorder

In Conway and Pleydell-Pearce's (2000) influential model of AM, which aims to account for variation in AM specificity, specific AM retrieval involves the reactivation of self-related knowledge from a hierarchical store. Executive processes evaluate the current contents of memory in relation to their relevance to current goals and are involved in terminating the search when the search goals (to retrieve a specific episode) are achieved. The ability to recall specific AMs is most commonly assessed using the Autobiographical Memory Test (AMT; Williams and Broadbent, 1986). Cued memories are considered specific if they contain unique temporal (specific event lasting < 1 day) or spatial (a specific location and arrangement of objects and people) episodic details. Reliability and validity of the AMT has been assessed across test re-test intervals ranging from 1 to 5 months (Raes et al., 2009b), and a study of 40,000 memories in young teenagers found that the AMT operates well over a wide range of scores and delivers a reliable continuous measure of overgeneral (i.e., non-specific) AM (Heron et al., 2012). The AMT reliably measures the single factor of AM specificity in both healthy (Heron et al., 2012) and clinical samples (Griffith et al., 2012).

AMT performance in psychological disorders is characterized by a tendency to retrieve a larger number of general relative to specific AMs in response to word cues. Based on this pattern of AMT performance, Williams (2006) and Williams et al. (2007) adapted Conway and Pleydell-Pearce's (2000) model to account for the overgeneral AM retrieval observed in emotional disorders (Williams et al., 2007; see also Ono et al., 2016). The Williams model emphasizes three critical mechanisms of cognitive maladaptation which, alone or in combination, contribute to overgeneral AM retrieval: *capture and rumination* (a tendency to perseverate on a negative, analytic form of memory processing)*, functional avoidance* (a tendency for memory search to be truncated to prevent full affective activation accompanying specific retrieval), and “*executive control*” (impairment in tracking of specificity of AMs), the so called CaRFAX model.

Overgeneral AM has consistently been associated with depression, PTSD, acute stress disorder, and other psychological disorders (Moore and Zoellner, 2007; Williams et al., 2007; Barry et al., 2021). Overgeneral AM has been found to lessen following natural remission or successful psychological treatment (Sutherland and Bryant, 2007; Ahern and Semkovska, 2017), suggesting that reduced specific AM retrieval may represent a state marker of psychological disorder. However, longitudinally, overgeneral AM also predicts PTSD symptoms (Bryant et al., 2007), while prepartum AM non-specificity (i.e., overgeneral AM) has been associated with more posttraumatic symptoms in those who had experienced a complicated pregnancy (Hauer et al., 2009). In an fMRI study of adolescents with or without documented experience of childhood maltreatment, AM-related brain activity was not a significant predictor of future psychosocial functioning, whereas, in those with maltreatment histories, greater overgeneral AM (assessed using the AMT) at baseline predicted reduced prosocial behavior at follow-up (Puetz et al., 2021).

Dysfunctional AM specificity has been suggested to be a consequence of trauma or depression (the scarring hypothesis, e.g., Stokes et al., 2004; Crane et al., 2014), or, alternatively, an antecedent trait, increasing the likelihood of developing depression or PTSD following a negative experience (the vulnerability hypothesis, e.g., Hauer et al., 2009). Overgeneral AM, assessed using the AMT is both associated with, and a predictor of, higher depressive symptoms at follow-up in clinical samples (Sumner et al., 2010; Warne et al., 2020; Hallford et al., 2021).

Understanding the factors that contribute to and potentially ameliorate dysfunction in AM specificity using the AMT and pharmacological tools could have implications for the treatment (or prevention) of several psychological disorders. In particular, if overgeneral AM has a causal role in symptom maintenance or deterioration (Hallford et al., 2021), (psycho)pharmacological treatments that reverse this dysfunction could be employed in the treatment of personality disorders (Startup et al., 2001), schizophrenia (Corcoran and Frith, 2003; Barry et al., 2019; Zhang et al., 2019), obsessive compulsion (Spinhoven et al., 2009), depression (Dalgleish and Werner-Seidler, 2014), and traumatic stress disorders (Schönfeld and Ehlers, 2006; Schönfeld et al., 2007).

### Experimental psychological strategies targeting AM specificity

A number of behavioral interventions potentially target memory specificity in neuropsychiatric disorders (e.g., life review/reminiscence therapy; Arean et al., 1993; Memory Specificity Training; Raes et al., 2009a) based on Williams's conceptualization of the causal role of overgeneral memory in emotional disorders (particularly depression) and in the latter case is specifically designed to reverse this cognitive phenotype. A meta-analytic review of the effect of Memory Specificity Training on symptoms in emotional disorders (Barry et al., 2019) found a substantial reduction in depressive symptoms, although these benefits appeared to be relatively short-lived. This might suggest that a maintenance form of this training is required in order to achieve lasting effects. In any case, memory specificity treatment development has significantly benefited from advances in our understanding of basic cognitive processes underlying symptoms of psychopathology (e.g., studies of the CaRFAX model), whereas progress in the development of pharmacological treatments targeting overgenerality has been slower. Understanding the neuropharmacological systems underlying memory specificity might therefore provide key insights into fruitful avenues for future pharmacological intervention development.

### Hypothalamic-pituitary-adrenal axis modulation and AM retrieval

Research on the neurobiology of stress and memory has provided significant insights into the neuropharmacology of AM by delineating the role of the HPA-axis in modulation of hippocampal-dependent (i.e., episodic) retrieval. Persistent hyperactivity of the HPA-axis due to prolonged stress, depression, or experiences of trauma can lead to significant structural and functional changes to key regions in emotional memory processing regions (i.e., the hippocampus, amygdala, and prefrontal cortex (PFC) (van Eijndhoven et al., 2009; Schmaal et al., 2016; Wellman and Moench, 2019), which may then influence episodic memory, including AM.

The hippocampus and amygdala are modulated by glucocorticoid and adrenergic regulators of the stress response system (Wang et al., 2013, 2014) and thought to critically support AM function. In AM retrieval, the amygdala has been shown to support emotional experience or the reinstatement of emotion at autobiographical retrieval (Bocchio et al., 2017; Ford and Kensinger, 2019), while hippocampal structures are thought to be responsible for accessing and integrating information into a spatiotemporally contextualized memory trace (for reviews see Cabeza and St Jacques, 2007; Sheldon and Levine, 2016; Sheldon et al., 2019). This integration (during encoding and retrieval) occurs across a broad network of brain structures involved in self-referential and emotional processing, autonoetic awareness, scene reconstruction and accuracy monitoring, which may at least partially explain why dysfunctional AM specificity has emerged as a transdiagnostic cognitive impairment in emotional disorders.

Abnormal HPA-axis functioning a common feature across psychological disorders including major depressive disorder (MDD; Stetler and Miller, 2011; Iob et al., 2020), PTSD (Steudte et al., 2011), schizophrenia (Aas et al., 2019) and borderline personality disorder (BPD; Drews et al., 2019). Hence, pharmacological research into the neurobiological correlates of depression and traumatic stress disorders (e.g., dysregulated HPA-axis functioning) may provide some clues as to the nature of the neurophysiological and neurotransmitter/neuromodulator disruption underlying overgeneral AM.

### Cortisol modulation

Stress-related episodic elevations in cortisol, analogous to those seen in depression (Bhagwagar et al., 2005; Herane-Vives et al., 2018) can be mimicked by experimentally elevating the stress-hormone cortisol, either endogenously (through stress induction procedures) or pharmacologically (through exogenous hydrocortisone administration). When cortisol elevation follows memory encoding this commonly results in improved delayed recall performance for emotionally salient aspects of memory (e.g., Smeets et al., 2008; Cunningham et al., 2021). However, when levels are endogenously or exogenously elevated prior to retrieval this has a deleterious effect on immediate recall (Smeets et al., 2008; Terfehr et al., 2011; Schwabe and Wolf, 2014; Merz et al., 2018). Both of these effects may further follow a non-linear (inverted U) dose-response relationship, producing seemingly discrepant findings and highlighting the complex interaction between episodic stress, hormonal concentrations, and memory processing.

### Modulation of membrane receptor targets

D-cycloserine (DCS), a partial agonist at the glycine binding site of the NMDA receptor, has long been thought to facilitate memory consolidation and retrieval (Quartermain et al., 1994), and has shown promising results in some studies when combined with behavioral (exposure-based) therapy (e.g., Inslicht et al., 2021; for reviews see Schade and Paulus, 2016; Mataix-Cols et al., 2017; Rosenfield et al., 2019). Typically, probing of monoaminergic, glutamatergic and oxytocin signaling can only be achieved pharmacologically.

Studies of serotoninergic modulation are of similar relevance to AM retrieval given the central role of serotonin in emotional processing and cognitive functioning (e.g., Hornboll et al., 2018; Knorr et al., 2019), as well as the proposed disturbance in serotoninergic functioning in some biological theories of depression (e.g., Ruhé et al., 2007) and serotonergic basis of its most common treatments. Acute tryptophan depletion has been shown to impair episodic retrieval (McAllister-Williams et al., 2002) yet evidence is mixed (e.g., van der Veen et al., 2006).

The dorsal hippocampus has been linked to an oxytocin-sensitive forebrain stress circuit, and as a central regulator (*via* suppression) of stress-induced neuroendocrine and molecular responses. As such, oxytocin may warrant investigation in AM retrieval dysfunction (Windle et al., 2004). In healthy volunteers, a single dose of oxytocin prior to learning (Herzmann et al., 2013) and prior to both learning and retrieval (Weigand et al., 2013) has been found to improve retrieval of negative emotional material. Both the HPA-axis and locus coeruleus (LC) -noradrenergic (NA) system are known to regulate the physiological stress response, yet the impact of LC-NA system dysregulation on AM specificity remains comparatively understudied. However, in similar studies of the acute effects of antidepressant treatments, monoaminergic modulators have been shown to improve positive information retrieval, in the absence of generalized improvements in cognitive performance (Harmer et al., 2003, 2008; Arnone et al., 2009). In extending these effects to clinical samples, Harmer et al. (2009) provided initial evidence that MDD patients may be more sensitive than healthy controls to episodic memory improvements following noradrenergic stimulation, as the acute administration of a noradrenaline reuptake inhibitor reboxetine resulted in an improvement in positive episodic retrieval in depressed patients only.

### The current review

Our understanding of the cognitive neuroscience of AM specificity has improved considerably in recent years (Barry et al., 2018). However, the neuropsychopharmacology of AM is less well understood. The goal of the current review is to systematically review findings from published placebo-controlled studies of the pharmacological modulation of AM specificity assessed using the AMT. We aim to address a gap in the AM with the aim of identify the most promising pharmacological systems for future AM research.

## Methods

### Preregistration and search strategy

The review methodology was preregistered on the PROSPERO prospective register of systematic reviews (reg. no: CRD42020199076). PsycInfo, PsycArticles, Ovid MEDLINE^®^, Embase, Scopus and Web of Science Core Collection databases, the Cochrane trial registry, and the OpenGrey and Open Access Theses and Dissertations databases were searched using the following terms: (Autobiographic^*^ OR ((personal^*^ or self^*^ or event^*^) adj2 memor^*^) OR ((real^*^life or personal^*^) adj1 (experience^*^ or episodic^*^ or event^*^))) AND ((tryptophan or Levotryptophan or l^*^tryptophan) OR (placebo^*^ or sham)). Note, “tryptophan” was included as a specific term since initial scoping searches failed to identify relevant tryptophan depletion studies known to the authors. The use of “placebo” was expected to capture placebo-controlled studies of pharmaceutical preparations. However, the term “sham” is typically used in tryptophan depletion studies, and inclusion of this term did indeed capture the TD studies. Proximity operators were adapted for each database. The ClinicalTrials.gov registry and World Health Organization International Clinical Trials Registry Platform were searched using the terms “*autobiographical OR episodic*” and “*memory*”. The Open Access Theses and Dissertations search was restricted to English language and doctorate theses only. The above listed searches were originally run on 2nd July 2020 and up-dated on 5th May 2022. One additional study was identified between these searches (Wong et al., 2021). No other restrictions (e.g., date restrictions) were placed on the search criteria. A pre-registered aim of the review was to perform a meta-analysis on effects of various pharmacological treatments on AM retrieval. However, upon review, we could not justify such a quantitative synthesis because of the small number of studies (usually only a single study) that examined each specific drug class, and the lack of a biological rationale for pooling studies across drug classes that targeted different neurotransmitter/neuromodulatory systems with different predicted directional effects on AM. As such, this aim could not be implemented in full, although a small number of methodologically homogeneous studies of hydrocortisone were combined to obtain a provisional effect size for this drug alone.

### Study selection

**Figure 1:**
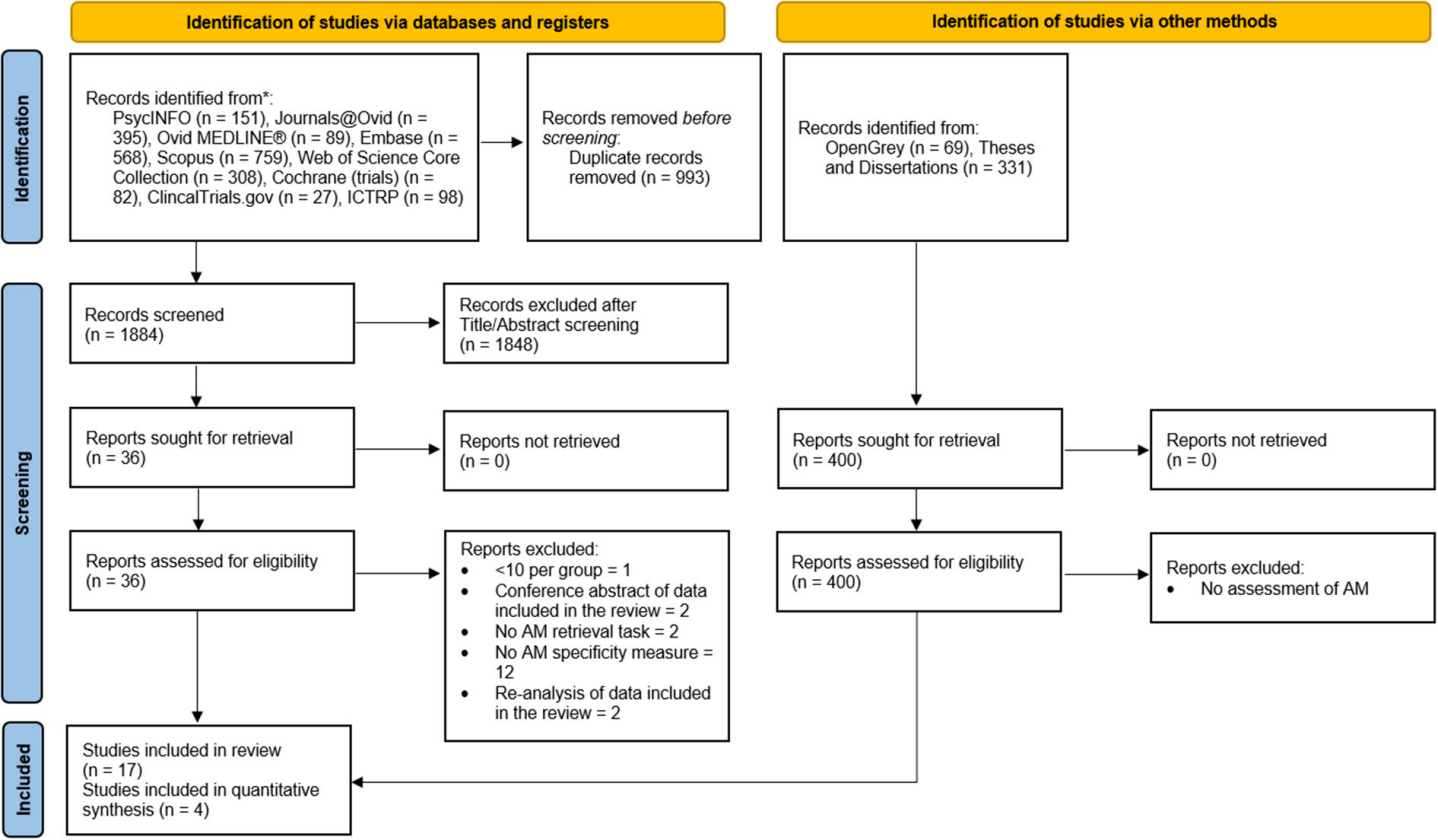
Page et al. (2021) flowchart of the study selection process.

Search, screening, and selection processes were conducted according to the Preferred Reporting Items for Systematic Reviews and Meta-Analyses (Page et al., 2021; Figure 1). Titles and abstracts were independently reviewed by the author (E.C) and another researcher (G.P). There were no disagreements on final study inclusions. Eligibility was restricted to randomized, placebo-controlled studies of adult participants, that including a pharmacological manipulation administered prior to AM retrieval. Eligibility was also restricted to studies where AM retrieval was elicited and scored for specificity using either the original or a modified version of the AMT to provide a continuous measure of the number of specific memories retrieved (Williams and Broadbent, 1986).

The included studies were diverse in terms of participant characteristics and the neurotransmitter/neuromodulator system(s) being targeted. Only within-subjects crossover studies that examined the effects of low dose hydrocortisone in healthy volunteers could be used to calculate a pooled effect size estimate (Buss et al., 2004; Schlosser et al., 2010; Wingenfeld et al., 2012a, 2013).

### Data extraction and study quality evaluation

The following study characteristics were independently extracted by two researchers (EC and GP): study design, nature of participant group(s), pharmacological treatment characteristics (drug, dose, route of administration), biological assessment of systemic drug levels, author-specified predictions on the effects of the drug on retrieval (i.e., improvement or reduction in specificity), AMT protocols, AMT performance (number of specific memories), other outcome and explanatory variables (Table 1). For outcomes included the pooled analysis of the effects of hydrocortisone in healthy volunteers, one author provided means and standard deviations since numerical values because these were not reported in the publication (Wingenfeld et al., 2012a). One study was not included in the pooled analysis because we were unable to obtain the means and standard deviations required (Fleischer et al., 2017). The methods sections of included studies were independently reviewed by EC and GP and the Cochrane risk of bias tool (Higgins et al., 2016) was used to evaluate methodological features of the reviewed studies.

**Table 1:** Characteristics of the 17 reviewed studies.

**References**	**Participant group**	**Design**	**N (%♀)**	**Mean age (SD)**	**Drug**	**Biological measurement(s)**	**Mechanism of action**	**Hypothesized effects**	**Drug admin (T0) to AMT (T1) (minutes)**	**Time to respond AMT**	**Outcome measure AMT (vs. placebo)**	**Other measures reported**
Buss et al. (2004)	Non-clinical	Crossover	22 (0%)	26.27 (0.9)	HC (10 mg) oral	Free salivary cortisol (nmol/l) Pre-drug intake (T0), +60 min (T1) (before memory testing)	GC receptor agonist	Specificity **↓** Free cortisol levels **↑**	60	60 s p/cue	Specificity **↓** Neutral cues only	• T0 to T1: Free cortisol levels **↑** • Mood and attention tests (data not reported)
Schlosser et al. (2010)	Non-clinical	Crossover (Between groups by diagnoses)	16 (50%)	33.31 (7.3)	HC (10 mg) oral	Not included	GC receptor agonist	Specificity **↓**	60	60 s p/cue	Specificity **↓** No interaction by cue valence	
	MDD – determined using the SCID-I and BDI		16 (50%)	34.88 (7.2)				Specificity ↔			Specificity **↔**	
Young et al. (2011)	Non-clinical Non-clinical	Crossover (Between groups by dose)	33 (52%) 33 (52%)	27.89 (5.8) 29.33 (7.8)	HC moderate dose (0.15 mg/kg; mean total dose 10.9 mg, SD 2.05) 2-min intravenous infusion HC high dose (0.45 mg/kg; mean total dose 31.8 mg, SD 8.74) 2-min intravenous infusion	Free plasma cortisol and corticosteroid binding globulin (μmol/L) Pre-infusion (T0), +15 (T1), +30 (T2), +45 (T3), +75 (T4) (before memory testing), +150 min (T5) (after testing)	GC receptor agonist	Specificity **↓** in dose-dependent manner (i.e., stronger effect in high dose) Free cortisol levels **↑**	75	No limit	Specificity **↔** Specificity **↓** No interaction by cue valence	• Baseline: Age, BMI, free plasma cortisol **↔** • T0 to T4, T5: Free plasma cortisol levels **↑** in high dose only • T0, T1, T2, T3, T4, T5: Total and free cortisol levels sex difference; ♀**↑** • AMT Latency **↔** • California Verbal Learning Test (Delis et al., 1987) **↔**
Wingenfeld et al. (2012a)	Non-clinical PTSD – determined using the Structured Clinical Interview for DSM-IV Axis I and II (SCID-I and II) and PDS	Crossover (Between groups by diagnoses)	65 (65%) 44 (86%)	31.7 (10.3) 30.09 (9.6)	HC (10 mg) oral	Not included	GC receptor agonist	Specificity **↓** Specificity **↓**/**↑** equally likely	30 - word list recall 60 - AMT	60 s p/cue	Specificity **↔** Specificity **↑** No interaction by cue valence	• Baseline: Mood via BDI^*^ • Baseline: General mental ability, LPS 3 and 4 of LPS German intelligence test **↔** • T0 (+24-h delay): Word list (free recall) non-clinical n=61 **↔**, PTSD n=36 **↑** • End of each visit: perceived situational stress and control VAS Qs: **↔**
Wingenfeld et al. (2013)	Non-clinical	Crossover (Between groups by diagnoses)	40 (100%)	32.9 (10.8)	HC (10 mg) oral	Not included	GC receptor agonist	Specificity **↓**	30 - word list recall 60 - AMT	60 s p/cue	Specificity **↓** No interaction by cue valence	• T0 (+24-h delay): Word list (free recall) **↔** • Visit 1 and 2 immediate, visit 1 and 2 + 24-h: Word Suppression Test (Terfehr et al., 2011) ° non-clinical HC *n* = 19, placebo n=21 **↔** ° BPD HC *n* = 33, placebo *n* = 34 **↔**
	BPD – determined using the SCID-I and II, BDI, PDS, CTQ		71 (100%)	28.2 (7.9)				Specificity **↑**			Specificity **↑** **Trend effect** (p=0.06) No interaction by cue valence	
Fleischer et al. (2017)	Non-clinical	Between subjects	28 (50%)	24.6 (3.7)	HC (10 mg) oral	Free salivary cortisol (nmol/l) Pre-drug intake (T0), +45 (T1), +75 (T2), +105 (T3) min	GC receptor agonist	Specificity **↓** Free cortisol levels **↑**	75	n/a	Specificity **↔** (Both groups)	• Baseline: Age^*^, BMI^*^, education^*^, smoking^*^, oral contraceptives^*^ • T0 to T1, T2, T3: Free cortisol levels **↑**
	Non-clinical		26 (62%)	24.3 (3.7)	Placebo							
Young et al. (2016) *Mifepristone results*	Non-clinical	Crossover	10 (0%)	28 (5)	Mifepristone/placebo (600 mg) oral (3 x 200-mg capsules at midnight, 3 placebo capsules at 5 am) Placebo/placebo (6 capsules; 3 at midnight, 3 at 5 a.m.) oral	Free plasma cortisol (μmol/L) Day before testing at 3 p.m. (T0) baseline sample, then during each test day from 7.30 a.m. to 10 a.m. at 30 min intervals (T1), (T2), (T3), (T4), (T5), (T6)	GC receptor antagonist	Specificity **↓** Free cortisol levels **↑**	240 (since last dose)	60 s p/cue	Specificity **↑** No interaction by cue valence	• Baseline (each visit): Anxiety, depression, and mood *via* STAI, HDRS, POMS • T1 to T6: Free plasma cortisol levels **↑** • Each visit: Word list (free recall) **↔** • fMRI to faces: ROI amygdala BOLD **↑** • fMRI to faces exploratory whole brain analyses
*Spironolactone results*					Spironolactone/ spironolactone (600 mg) oral (6 x 200-mg capsule; 3 at midnight, 3 at 5 am) Placebo/placebo (see above)		MC receptor antagonist				Specificity **↓** No interaction by cue valence	
Fleischer et al. (2015)	Non-clinical	Crossover (Between groups by diagnoses)	67 (100%)	30.2 (13.1)	Fludrocortisone (0.4 mg) oral	Not included	MC receptor agonist	Specificity **↑**	90	60 s p/cue	Specificity **↔** (All groups)	• Baseline: Age^*^, education^*^, BMI^*^, smoking^*^, oral contraceptives^*^
	BPD		37 (100%)	24.8 (5.8)				Specificity **↓**				
	MDD		24 (100%)	35.6 (14.8)				Specificity **↑**				
Carvalho et al. (2006)	Non-clinical	Between subjects	32 (56%)	23.0 (0.4)	Sertraline (50 mg) oral	Not included	Selective 5-HT reuptake inhibition	Specificity **↑**	360	60 s p/cue	Specificity **↔** (All groups)	• Baseline: Mood via BDI, age, sex, education **↔** • AMT Latency **↔** ° Visit day + 7-days: Emotional story with slides memory test (surprise) **↔**
	Non-clinical		32 (53%)	22.5 (0.4)	Bupropion (150 mg) oral		NE–DA reuptake inhibition	Specificity **↑**	180			
	Non-clinical		35 (51%)	23.1 (0.5)	Placebo				180 or 360			
Park et al. (1994)	Non-clinical	Crossover	12 (0%)	29 (SD not reported)	L-alanine (2.75 g), L-arginine (2.45 g), L-cysteine (1.35 g), L-glycine (1.6 g), L-histidine (1.6 g), L-isoleucine (4 g), L-leucine (6.74 g), L-lysine (5.5 g), L-methionine (1.5 g), L-phenyalanine (2.85 g), L-proline (6.1 g), L-serine (3.45 g), L-threonine (3.45 g), L-tyrosine (3.45 g), L-valine (4.45 g) Control drink contained the depletion mixture +(1.15 g) L-tryptophan, oral (in 300 ml water)	Total and free TRP concentration in plasma (μmol/L) Pre-drug intake (T0) and +235 min (T1) (before memory testing)	5-HT depletion	Not reported	235–330	Not reported	Specificity **↔**	• T0 to T1: Total plasma and free TRP levels **↓** • Mood *via* VAS **↔** • Cambridge Neuropsychological Test Automated Battery ° Paired Associates learning **↓** ° Visual Discrimination learning **↓** • Tower of London; thinking time **↓**
Haddad et al. (2009)	Non-clinical, remitted depression – at least one lifetime major depressive episode, but not met criteria for previous 6 months, determined using SCID-I	Between subjects	12 (100%)	28.3 (10.3)	L-isoleucine (4.2 g), L-leucine (6.6 g), L-lysine monohydrochloride (4.8 g), L-methionine (1.5 g), L-phenylalanine (2.9 g), L-tyrosine (3.4 g), L-threonine (3.0 g), L-valine (4.8 g), oral (in 200 ml water)	Mean % decrease in total TRP in plasma (μmol/L) Pre-drug intake (T0) and +300 min (T1) (before memory testing)	5-HT depletion	Specificity **↓** Total plasma TRP levels **↓**	300	20 s p/cue	Specificity **↓***(Diff from no drink at baseline)* Negative cues only	• Baseline: Mood via BDI, HDRS **↔** • Baseline: total TRP levels **↔** • Low baseline Number Generation Task (NGT) score associated with T1 Specificity **↓** • T0 to T1: Total plasma TRP levels **↓** • T0 to T1: NGT **↔** • T0 to T1: Mood via HDRS-m, BDI, POMS, VAS scales **↔** ° T1: Auditory Verbal Learning Test (Rey, 1964) immediate recall scores only **↓** T1: Remember–Know Test (Anderson, 1968) accuracy and mean RT **↔**
	Non-clinical, remitted depression – see above definition		12 (100%)	24.6 (5.3)	Control drink contained the depletion mixture +(2.0 g) of L-tryptophan, oral (in 200 ml water)						Specificity **↔**	
Alhaj et al. (2012)	Non-clinical, family history of MDD – using the MINI and BDI, and adapted Family History Research Diagnostic Criteria (Endicott, 1978)	Crossover	19 (95%)	21.4 (2)	L-alanine (2.75 g), L-arginine (2.45 g), L-cysteine (1.35 g), L-glycine (1.6 g), L-histidine (1.6 g), L-isoleucine (4 g), L-leucine (6.75 g), L-lysine monohydrochloride (4.45 g), L-methionine (1.5 g), L-phenylalanine (2.85 g), L-proline (12.2 g), L-serine (3.45 g), L-threonine (3.25 g), L-tyrosine (3.45 g) and L-valine (4.45 g), oral (in 300 ml water) Control drink contained the depletion mixture +(1.15 g) of L-tryptophan, oral (in 300 ml water)	Total and free TRP concentration in plasma (μmol/L), and ratio of tryptophan to the sum of other large neutral amino acids Pre-drug intake (T0) and +300 min (T1) (before memory testing)	5-HT depletion	Specificity **↓** Total plasma TRP levels **↓**	300	30 s p/cue	Specificity **↔**	• T0 to T1: Total plasma TRP levels **↓** ° T0 to T1: Mood via HDRS-m, VAS, POMS **↔**
Wingenfeld et al. (2012b)	Non-clinical	Crossover (Between groups by diagnoses)	18 (83%)	35.3 (10)	Yohimbine (5 mg) oral	Salivary alpha amylase, HR, BP Pre-drug intake (T0), +15 (T1), +75 min (T2) (not specified whether before/after memory testing)	alpha-2 adrenergic antagonist	Specificity **↑**	60	60 s p/cue	Specificity **↔** (Both groups)	• Baseline: General mental ability via LPS 3 and 4 of LPS German intelligence test **trend difference** (*p* = 0.051) • T0 to T1, T2: **trend effect** (p=0.08) **↓**, BP **↑**, HR **↔** • T2: Word Suppression Test (Terfehr et al., 2011) **↔** • + 24-h delay: Word list (free recall) **↑**
	MDD – determined using the SCID-I, BDI, CTQ		20 (80%)	35.1 (9.9)				Specificity **↑** (Stronger effect than non-clinical sample)				
Kuffel et al. (2014)	Non-clinical MDD – SCID-I, BDI	Crossover (Between groups by diagnoses)	20 (70%) 20 (70%)	30.8 (9.2) 30.8 (9.2)	Clonidine (0.15 mg) oral	Salivary alpha amylase, BP Pre-drug intake T0, +15 (T1), +75 min (T2) (not specified before/after memory testing)	alpha-2 adrenergic agonist	Specificity **↓** Salivary alpha amylase **↓**, BP **↓**	60	60 s p/cue	Specificity **↔** (Both groups)	• Baseline: Mood via BDI, MDD **↑** • T0 to T1, T2: Salivary alpha amylase **↓**, BP **↓** • T2: Word Suppression Test (Terfehr et al., 2011) **↔** • T0 (+ 24-h delay): Word list (free recall) **↓**
Cardoso et al. (2014)	Non-clinical	Crossover	17 (0%)	23.1 (3.5)	Oxytocin (24 IU) intranasal	Not included	Increasing bioavailability of oxytocin	Specificity **↑** (No prediction of dose dependent effects)	110	No limit	Specificity **↑** Overgeneral **↓** No interaction by cue valence	• Final visit + M=13.57 (SD = 7.25) days: ratings of AMT memories transcriptions on Likert 1-9, positive ratings **↑** in 24 IU dose only
					Oxytocin (48 IU) intranasal						Specificity **↔**	
Wong et al. (2021) (Study 1)	Non-clinical	Crossover	48 (50%)	23.7 (3.52	Oxytocin (24 IU) intranasal	Not included	Increasing bioavailability of oxytocin	Specificity **↓**	38	Not reported	Specificity **↔**	• BDI-II • Vividness ° EEG analysis, not reported
Wong et al. (2021) (Study 2)	Non-clinical	Crossover	63 (50.79%)	24.6 (4.22)	Oxytocin (24 IU) intranasal	Not included	Increasing bioavailability of oxytocin	Specificity **↓**	90	Not reported	Specificity **↔**	• BDI-II • Vividness **↔** • Eye-tracking analysis, not reported
Chen et al. (2020)	Non-clinical Non-clinical	Between subjects	20 (50%) 20 (55%)	23.45 (4.1) 22.7 (3.3)	D-cycloserine (250 mg) oral Placebo	Not included	NMDA partial agonist	Specificity **↑**	180	60 s p/cue	Specificity **↑** (Persisted after 24-h) No interaction by cue valence Specificity **↔**	• Baseline: Age, gender, education, BDI, STAI-T, EPQ, Verbal IQ (STW) **↔** • T0 to T1: Mood and subjective effects via STAI-S, PANAS, BFS, VAS **↔** • T1 for DCS vs. placebo: Facial Expression Recognition Task (FERT) **↔**, Emotional Categorization Task positive word category only **trend effect** (*p* = 0.058) **↑**, Emotional Recall Task positive word recall only **↑**, Emotional Recognition Memory Task (EREC) **NS**, Facial Dot-Probe Task **↔** ° 24-h delay: STAI-S, PANAS, BFS, VAS **↔**, FERT **↔**, EREC **↔**, AMT Specificity **↑**

* Indicates statistically significant (*p* ≤ 0.05) between group differences at baseline. **↓** = sig. reduction, **↑** = sig. increase, and **↔** = no sig. difference indicate statistically significant (*p* ≤ 0.05) drug vs. placebo effects on AMT performance and other assessments.

Hydrocortisone (HC), glucocorticoid (GC), mineralocorticoid (MC), serotonin (5-HT), noradrenaline (NE), dopamine (DA), tryptophan (TRP), international unit (IU), N-methyl-D-aspartate (NMDA), Heart rate (HR), blood pressure (BP), seconds (s), Body Mass Index (BMI), Posttraumatic stress disorder (PTSD), major depressive disorder (MDD), borderline personality disorder (BPD). Structured Clinical Interview for DSM disorders (SCID), Diagnostic and Statistical Manual (American Psychiatric Association, 2000), Mini-International Neuropsychiatric Interview (MINI; Sheehan et al., 1998; Lifetime version IDDL; Zimmerman and Coryell, 1987). Beck Depression Inventory (BDI; Beck et al., 1961; Beck et al., 1994), Posttraumatic Stress Diagnostic scale (PDS; Foa, 1995), Childhood Trauma Questionnaire (CTQ; Bernstein et al., 2003), Leistungsprufsystem (LPS; Horn, 1983), Bond-Lader Visual Analog Scales (VAS), Hamilton Depression Rating Scale (HDRS; Hamilton, 1960), Profile of Mood States (POMS; McNair et al., 1971, 1992), Spielberger Trait Anxiety Inventory (STAI-T; Spielberger et al., 1971), Eysenck Personality Questionnaire (EPQ; Eysenck and Eysenck, 1976), Positive and Negative Affect Schedule (PANAS; Watson et al., 1988), the Spot-the-Word task (STW; Baddeley and Emslie, 1993).

### Effect size determination

Effect sizes on the number of specific AMs generated after drug and placebo administration were calculated by dividing mean differences by pooled SDs and applying a correction for small sample bias (i.e., Hedges' g). A pooled ES was determined *via* a random effects model using maximum likelihood estimation for the four studies that used low dose hydrocortisone in healthy volunteers using SPSS (IBM Corp. Released 2020. IBM SPSS Statistics for Windows, Version 27.0. Armonk, NY: IBM Corp).

## Results

### Study and sample characteristics

The literature search yielded a total of 17 studies from 11 publications. A total of *n* = 986 participants (*mean age* = 27.65, *SD* = 4.33) were included across all reviewed studies. This included data from six studies of clinical participants involving *n* = 108 participants with a diagnosis of BPD, *n* = 80 with MDD, and *n* = 44 with PTSD (Schlosser et al., 2010; Wingenfeld et al., 2012a,b, 2013; Kuffel et al., 2014; Fleischer et al., 2015; see Table 1). Clinical participants were commonly recruited via psychiatric centers and the comparator control participants via local advertising. Diagnoses in studies of clinical participants were established using the Structured Clinical Interviewing for the DSM-IV which were also used to screen and exclude control participants (i.e., based on current or historic psychiatric diagnoses) in the studies of clinical groups. Two studies examined participants who were in remission but at risk of MDD (*n* = 43) (Haddad et al., 2009; Alhaj et al., 2012), while the remaining nine studies only included healthy volunteers (*n* = 711) (Park et al., 1994; Buss et al., 2004; Carvalho et al., 2006; Young et al., 2011, 2016; Cardoso et al., 2014; Fleischer et al., 2017; Chen et al., 2020; Wong et al., 2021).

### Methodological characteristics

#### General study features

Key design and methodological features of the included studies are summarized in Table 1. Common methodological strengths included double blinding of drug administration; (except for Wingenfeld et al., 2013; only participant-blinded), and counterbalancing of drug conditions in within subject crossover designs; (except for Haddad et al., 2009). Inclusion and exclusion criteria were generally well reported in all of the reviewed studies. With the exception of Haddad et al. (2009) no other studies explicitly reported on the absence or presence of missing data in the AMT. Using information published in each study, it was determined that thirteen publications had no missing AMT data. However, missingness could not be determined using the information provided in the three remaining included studies (Park et al., 1994; Carvalho et al., 2006; Fleischer et al., 2015).

#### Autobiographical Memory Test

The AMT protocol and scoring procedures were generally well-described in all of the reviewed studies. Small inconsistencies in memory scoring procedures may be a minor methodological weakness to the overall quality of the reviewed research, as the type and frequency of details required for a memory to be rated as “specific” varied slightly across studies. Only one study indicated that rating of AM responses was blind (Young et al., 2016). Ten studies reported using independent raters of participant responses (in most cases using a subset of memory descriptions; Buss et al., 2004; Schlosser et al., 2010; Young et al., 2011, 2016; Wingenfeld et al., 2012a; Cardoso et al., 2014; Fleischer et al., 2015, 2017; Chen et al., 2020; Wong et al., 2021). Of the ten studies using independent rating, seven reported indices of inter-rater agreement of AM scoring which were generally high (0.75–0.97; Buss et al., 2004; Schlosser et al., 2010; Young et al., 2011, 2016; Cardoso et al., 2014; Chen et al., 2020; Wong et al., 2021).

#### Assessment of systemic drug levels

Nine studies included biochemical assays of drug levels (Park et al., 1994; Buss et al., 2004; Haddad et al., 2009; Young et al., 2011, 2016; Alhaj et al., 2012; Wingenfeld et al., 2012b; Kuffel et al., 2014; Fleischer et al., 2017; see Table 1). In all of these studies, the expected changes in drug (or amino acid) level were observed.

#### Risk of bias

Sixteen of the included studies were evaluated to have a low risk of bias based in items from the commonly used Cochrane risk of bias tool. One study was found to have a moderate risk of bias due to not including details on participant or experimenter blinding to placebo vs. drug treatment (Wingenfeld et al., 2013). A summarized figure of the risk of bias assessment is provided in the Supplementary material. It should be noted that none of the studies were “clinical trials” and most pre-date the increasingly common practice of pre-registration of experimental studies.

Two studies were funded by industry sponsors, yet in both cases the authors reported no influence of sponsorship on either study (Wingenfeld et al., 2012b; Young et al., 2016). The contributions of a single author to one study (C. Otte; Fleischer et al., 2015), and of two authors in another study (K. Wingenfeld and B. Lowe; Kuffel et al., 2014) were supported by industry sponsors. However, it should be reiterated that none of the studies were clinical trials investigating the efficacy of a medicinal product for a specific indication.

### Synthesis of study findings

#### Pharmacological targets

Studies targeted the glucocorticoid (k = 6), mineralocorticoid (k = 2), monoaminergic (k = 6), oxytocinergic (k = 2), and glutamatergic systems (k = 1; Table 1). It is noteworthy that no studies on cholinergic or GABAergic modulators, which include the classic amnestic agents (anticholinergics and benzodiazepines, respectively), were identified in the search.

#### Corticosteroid modulation: Glucocorticoid modulating compounds

Of the seven studies targeting glucocorticoid modulation, five had methodological similarities, particularly in relation to the use of a single low oral dose (10 mg) of hydrocortisone (Buss et al., 2004; Schlosser et al., 2010; Wingenfeld et al., 2012a, 2013; Fleischer et al., 2017). Among these, three non-clinical studies reported statistically significantly impairment of AM specificity relative to placebo (Buss et al., 2004; Schlosser et al., 2010; Wingenfeld et al., 2013). However, this effect was not observed in two other non-clinical studies (Wingenfeld et al., 2012a; Fleischer et al., 2017). In the only study of intravenous hydrocortisone in non-clinical participants, a high dose (0.45 mg/kg) led to a reduction in the percentage of specific AMs and simultaneous increase in categorical (i.e., non-specific) AM retrieval relative to placebo (Young et al., 2011; see Table 1; this latter effect was also seen with a three-fold lower dose).

Three of these five low-dose oral hydrocortisone studies were conducted in participants with a psychiatric disorder (Schlosser et al., 2010; Wingenfeld et al., 2012a, 2013). In a small study with MDD patients, no effect of hydrocortisone was found (Schlosser et al., 2010), although a larger study with PTSD patients showed significantly higher AM retrieval relative to placebo (Wingenfeld et al., 2012a; see Table 1). A similar specificity-enhancement effect was observed in a larger sample of BPD patients although this did not reach statistical significance (Wingenfeld et al., 2013; see Table 1). In both cases, the authors suggest this result may be due to improved reactivity to exogenous cortisol in these particular clinical populations, but this cannot be confirmed in either study due to a lack of additional measures of HPA axis functioning (e.g., basal cortisol levels).

In a within-subjects crossover study of glucocorticoid *antagonism* by mifepristone on AM retrieval (in a non-clinical sample), reduced glucocorticoid receptor activation significantly *increased* the percentage of specific AMs retrieved relative to placebo, albeit in a small, all-male sample (Young et al., 2016; see Table 1).

#### Effect size for AM specificity effect of low-dose hydrocortisone

**Figure 2:**
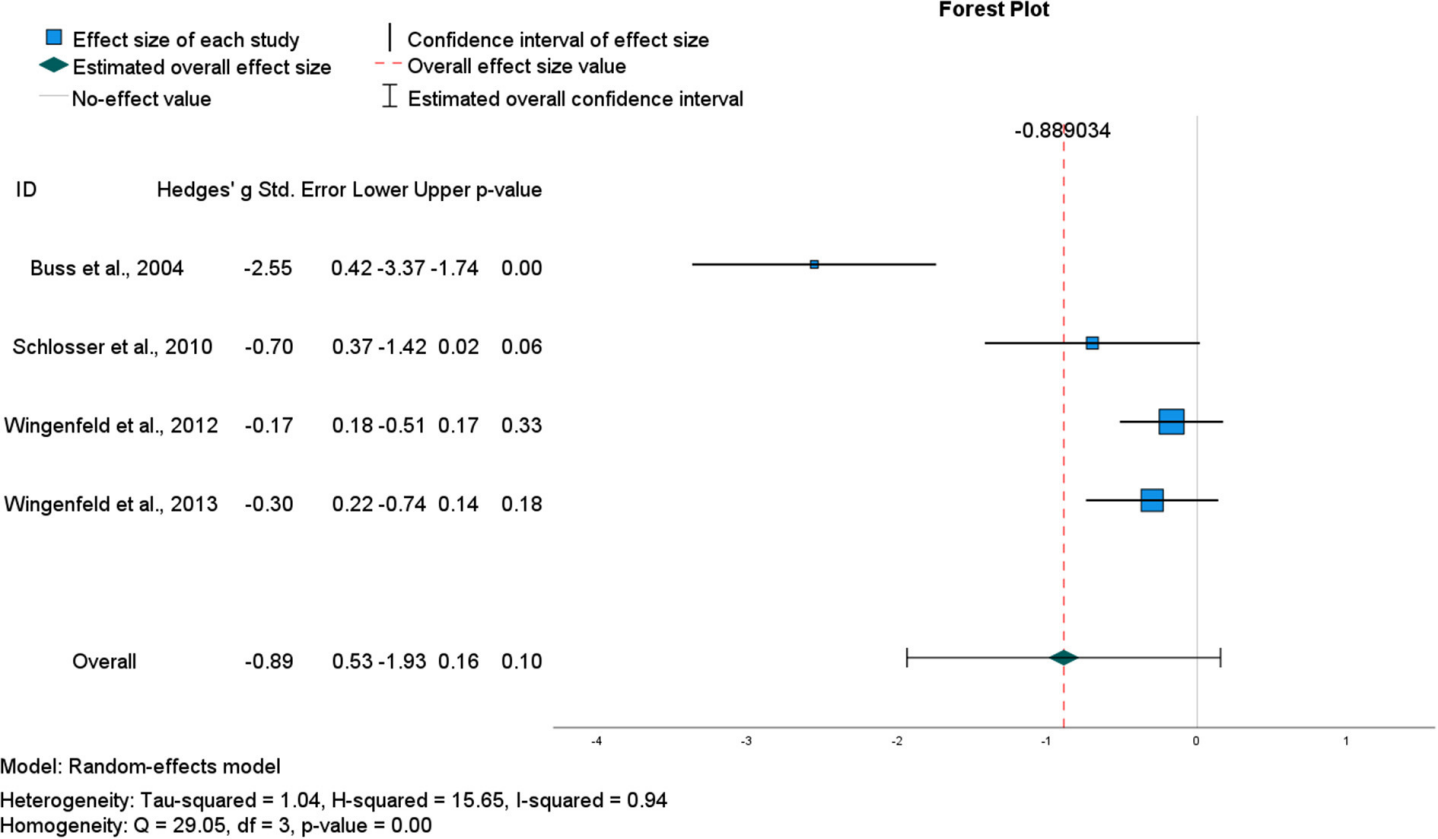
Forest plot for pooled studies of the (acute) effect of oral hydrocortisone (10 mg) administration in healthy participants on the number of specific AMs recalled.

**Figure 3:**
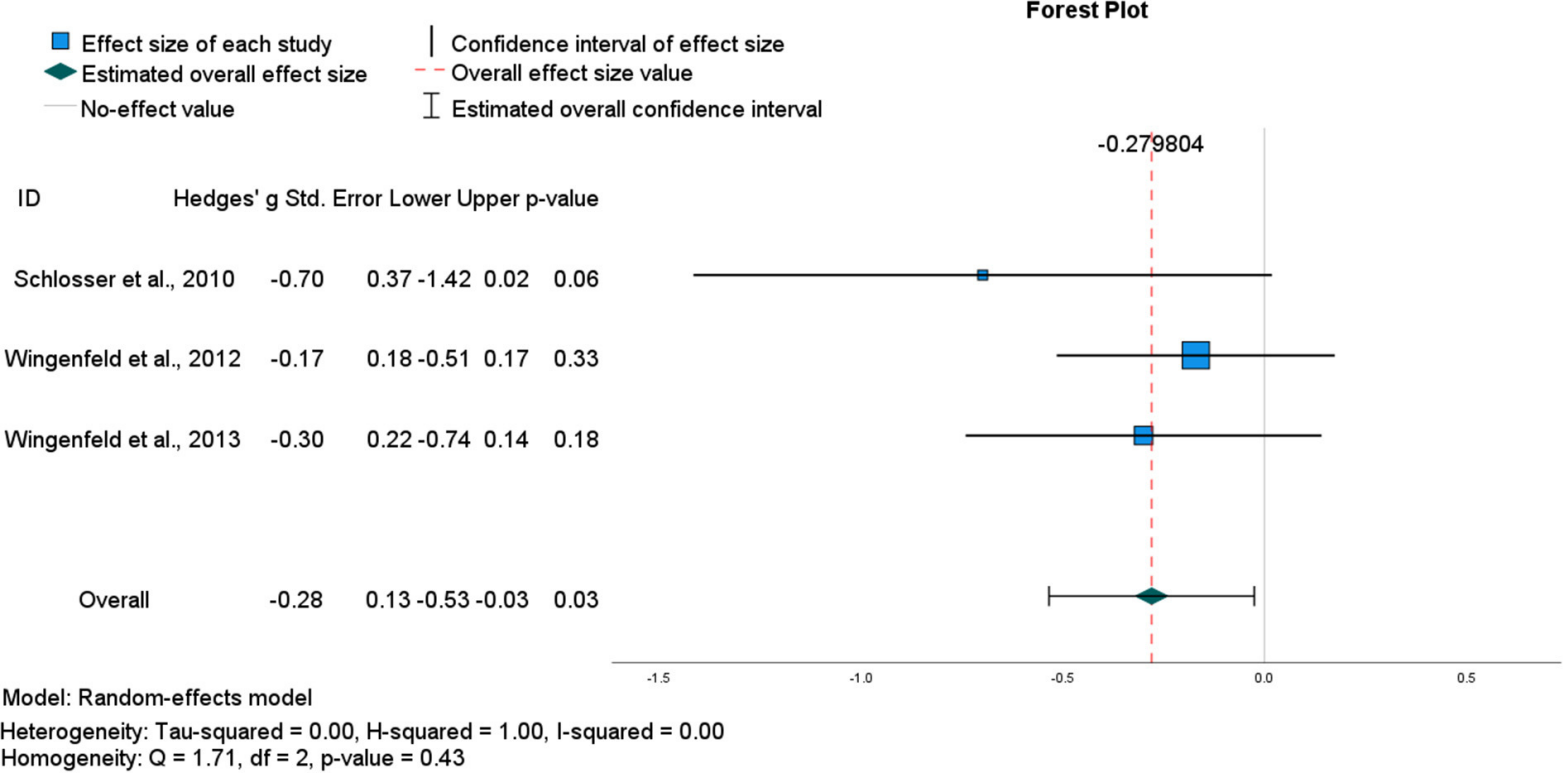
Forest plot for final pooled studies of the (acute) effect of oral hydrocortisone (10 mg) administration in healthy participants on the number of specific AMs recalled.

The general impairing effect of hydrocortisone on retrieval specificity among healthy participants in the studies described above is consistent with the notion of divergent acute effects of glucocorticoids on encoding vs. retrieval (Roozendaal, 2003). Data from four within-subject crossover studies using the same oral dose of hydrocortisone (10 mg) and following the same AMT protocol were pooled to determine an overall effect size (Buss et al., 2004; Schlosser et al., 2010; Wingenfeld et al., 2012a, 2013; see Table 1). Although the pooled ES was large, it was estimated with low precision [*g* = −0.89, CI (−1.93, 0.16)] and hence, was not significant (*p* = 0.10) (Figure 2). Heterogeneity statistics indicated a very large degree of between study heterogeneity (*I*^2^ = 94%), consistent with the small number of studies and the single outlier study (Buss et al., 2004). Removing Buss et al. (2004) reduced heterogeneity (*I*^2^ = 0%) and the aggregate ES [*g* = −0.28, CI (−0.53, −0.03)], (*p* = 0.03) (Figure 3). However, in this second analysis, the general impairing effect of hydrocortisone on healthy participants' AM retrieval specificity was statistically significant.

#### Mineralocorticoid receptor preferring drugs

Two studies have investigated drugs with a preference for mineralocorticoid receptors. Fludrocortisone (0.4 mg, oral), a selective mineralocorticoid receptor agonist had no effect on specificity in healthy women or in women with MDD and BPD (Fleischer et al., 2015). However, in a small sample of healthy men, AM specificity was impaired during mineralocorticoid receptor antagonism using spironolactone (Young et al., 2016; see Table 1).

#### Monoaminergic modulation

Wingenfeld et al. (2012b) hypothesized that blocking the presynaptic alpha-2-adrenoceptor to increase NA activity using a low-dose (5 mg) yohimbine would improve AM memory performance at retrieval. Harmer et al. (2009) predicted larger effects in MDD patients vs. non-clinical controls following reboxetine. Although a general improvement was observed on delayed (24 h) free recall of a wordlist in healthy and depressed participants, the effect was stronger in MDD. Yohimbine had no effect on AM specificity at retrieval in healthy or depressed participants.

Similarly, a single dose of the NE–DA reuptake inhibitor bupropion or the selective serotonin reuptake inhibitor sertraline produced no detectable effects on AM specificity in non-clinical volunteers (Carvalho et al., 2006; see Table 1). Kuffel et al. (2014) proposed a potential upregulation of alpha-2 receptors in depressed participants and predicted that clonidine, an alpha 2 agonist, would impair specificity in both non-clinical and MDD samples. However, they found no treatment effect on AM specificity.

Three studies have assessed the effect of low-dose tryptophan depletion on AM specificity and included analysis of plasma concentrations which confirmed that the treatment (i.e., acute tryptophan depletion) was successful in all three. One reported a significant reduction in specificity due to the treatment but in negative cue words only (Haddad et al., 2009), while the other two found no effects of treatment on AM specificity (Park et al., 1994; Alhaj et al., 2012; see Table 1).

#### Oxytocin and glutamatergic modulation

A moderate dose of oxytocin (intranasal) improved AM specificity relative to the high dose and placebo in a single, small-scale, study of healthy men (Cardoso et al., 2014; see Table 1). Wong et al. (2021) examined the effect of a moderate oxytocin dose on AM specificity in two studies of healthy volunteers and found no effect on the number of specific or overgeneral AMs retrieved in either study (see Table 1).

In the only identified placebo-controlled study of glutamatergic regulation of AM, Chen et al. (2020) reported that pre-retrieval d-cycloserine improved AM specificity, and this effect persisted for 24 h following drug administration (see Table 1). No concurrent effects were reported on subjective mood, emotional facial expression recognition, or word recall.

## Discussion

This review provides the first synthesis of studies examining pharmacological modulation of AM specificity. The systematic search identified a relatively small number of studies in this area, commensurate with our limited understanding of the neuropsychopharmacology of AM retrieval. We hope that this review will motivate further research on this topic, which has potential implications for our understanding of, and the development of treatments for, a range of psychological disorders. Given the included studies' diversity in terms of the neurotransmitter or neuromodulatory systems targeted, it was generally not possible to quantitatively synthesize the effects. A critical mass of studies on each primary drug group discussed here will allow researchers to undertake such quantitative analyses in the future.

Where a preliminary quantitative synthesis was possible (on the effects of low dose oral hydrocortisone in non-clinical participants), study effects were heterogeneous, largely due to an outlying single study (Buss et al., 2004). Following sensitivity analysis, the pooled effect size was modest yet statistically significant, and this impairing effect of hydrocortisone in non-clinical participants was in the direction predicted by each study's authors (impaired specificity). Lower AM specificity following hydrocortisone administration is consistent with literature that reports broad retrieval impairments with both endogenous and exogenously elevated cortisol, for example, stimulus-response tasks in rodents and in emotional word list, and probabilistic classification learning in humans (de Quervain et al., 2007; Atsak et al., 2016; Zerbes et al., 2019). The current results tentatively suggest that low dose hydrocortisone could be used to model dysfunctional AM specificity in non-clinical participants.

In the single study using intravenous hydrocortisone in healthy volunteers, only a high dose (~31.8 mg/kg) produced the expected increase in plasma cortisol, corticosteroid binding globulin concentrations, and impairment of AM specificity (Young et al., 2011). The importance of including biological indicators of treatment efficacy is underscored by Young et al.'s (2011) study, and so where possible, physiological baseline differences relevant to the neuromodulator system under assessment should be recorded in future studies (e.g., baseline cortisol levels; Young et al., 2011) and any intended elevation in cortisol by drug verified biologically. Based on these findings, future research should aim to replicate these enhancing and impairing effects at the suggested doses and extend them to include female and clinical samples. The combination of sex and sex-hormone levels can influence the effect of exogenous hydrocortisone on retrieval of aversive memories in healthy volunteers (e.g., Hennessy et al., 2022), and sex differences are well-established in PTSD (for reviews see Li and Graham, 2017; Kornfield et al., 2018) and episodic memory (Ertman et al., 2011; Soni et al., 2013).

In a large study of PTSD patients, most of whom reported repeated experiences of childhood trauma, low dose (10 mg) hydrocortisone resulted in significantly improved specific AM retrieval –opposite to the effect seen in healthy participants (Wingenfeld et al., 2012a). An enhancing effect of exogenous hydrocortisone on AM specificity was also observed in BPD patients (Wingenfeld et al., 2013; see Table 1), implying a potential common glucocorticoid-mediated disorder in AM retrieval (and possible treatment target) across these two disorders. In a similar placebo-controlled crossover study with healthy controls, PTSD and BPD patients, and using low dose hydrocortisone, the same research team found that in both PTSD and BPD patient groups, childhood trauma and symptom severity were negatively correlated with functional connectivity between regions associated with successful autobiographical retrieval (the hippocampus and dorsomedial PFC) (Metz et al., 2019).

Considering this evidence, it is possible that the distinct effects of hydrocortisone in PTSD and BPD vs. healthy volunteers reflects a developmental-stress-mediated change in glucocorticoid receptor functioning in chronic and complex PTSD and BPD, the neuropsychological symptoms of which can be partially remediated by the administration of exogenous cortisol. PTSD with and without MDD is associated with lower daily cortisol output relative to non-trauma exposed controls, with general trauma exposure associated with enhanced HPA feedback (Morris et al., 2012; Rauch et al., 2020). Moreover, stress-induced (endogenous) elevation in cortisol has no such effect on AM specificity in both BPD patients and healthy controls (Duesenberg et al., 2019).

It is likely that glucocorticoid receptor expression/sensitivity is adaptively downregulated in the presence of chronically elevated cortisol; potentially explaining the opposing effect of elevated cortisol in clinical samples vs. healthy volunteers. In the studies reviewed, BPD and MDD patient volunteers were overrepresented relative to those with PTSD. No manipulation effects were observed on the AMT for MDD patients, however, the potential effect of low dose hydrocortisone on AMT performance in MDD was not investigated in the reviewed research and may therefore represent a novel avenue for future study. Childhood (and adult) psychological trauma is associated with dysregulated cortisol secretion and blunted cortisol responding, as well as dendritic atrophy (McGowan et al., 2009; Wellman and Moench, 2019), and experience of childhood trauma appears to induce glucocorticoid resistance in those with depression (Nikkheslat et al., 2020). Therefore, this subgroup of MDD patients (with childhood trauma exposure-induced glucocorticoid receptor downregulation) may selectively benefit from a pre-retrieval low dose hydrocortisone administration to enhance AM specificity similar to those with PTSD and BPD.

A U-shaped dose-response relationship was reported for the effects of oxytocin on AM specificity (Cardoso et al., 2014), as only the moderate dose had an impact (positive) on specificity. The authors suggest that oxytocin may enhance self-referential processing with downstream beneficial effects on AM specificity, and that diminished effects at higher doses may be due to partial occupation of arginine vasopressin receptors by oxytocin (Manning et al., 2008, 2012). In a larger sample of men who underwent Pavlovian fear conditioning, a subsequent 24 IU dose of oxytocin was shown to enhance fear memory extinction, and this was associated with a general upregulation of PFC responding and downregulation in the amygdala (Eckstein et al., 2015). Thus, moderate doses of oxytocin appear to *generally* enhance emotional memory retrieval processes. It is therefore unclear whether studies of oxytocin will provide any special insights into the neuropharmacology of AM.

Chen et al. (2020) found that in healthy volunteers, a single pre-retrieval administration of d-cycloserine (250 mg) improved AM specificity, and this improvement persisted for at least 24 h. Research on emotional and declarative memory has recently focused on the relationship between stress and stress-related hormonal and neurochemical mediators (particularly glucocorticoids) and increased glutamatergic transmission in the PFC. This stress-induced increase in glutamatergic function has been implicated in beneficial effects on cognition and emotional processes (e.g., enhanced memory consolidation), yet also dysfunction in such processes through over-activation of the stress response system in neuropsychiatric disorders (Popoli et al., 2012; Wellman and Moench, 2019).

### Strengths and limitations of the reviewed studies

There are common methodological strengths across the reviewed studies despite their limited number and the range of neuromodulator systems targeted. Overall, the Cochrane Risk of Bias tool suggested that the studies had a low risk of bias. On the other hand, our risk of bias assessment was limited because AMT-specific methodological details were not captured by the Cochrane tool. While these specific methodological aspects were generally strong, no study was pre-registered. Preregistration and standardization of AMT scoring procedures will be key to providing like-for-like comparisons of drug effects in this field going forward.

The overall numbers of clinical participants contributing data to the current review was small. Furthermore, considering the limited available data for the reviewed drug class categories, it is not yet possible to determine the effect (size) of each drug class on AM specificity or possible moderation by clinical diagnoses. In general, based on the limited data, hydrocortisone, d-cycloserine, and oxytocin in particular appear to warrant further investigation in healthy volunteers. Pre-retrieval hydrocortisone should also be investigated to potentially improve specificity in clinical populations, particularly PTSD and BPD patients. While “positive” findings with these drugs should motivate replication and extension, further research on the other drug groups outlined here should not be foreclosed simply because of a small number of null results. As noted above, sex/sex hormones play a critical role in emotional memory, and critically, in the sensitivity to drug effects on emotional memory. An absence of evidence for pharmacological modulation may simply reflect an inability of some study designs to detect such modulation because of a failure to account for sex (hormones). As such, future studies should incorporate sex as an explanatory factor into their study design (potentially as a moderator in the pre-registered data analysis plan).

Where possible, future pharmacological research probing the neurobiology of AM specificity (e.g., using low dose hydrocortisone) should assess baseline endogenous neuromodulator functioning. Significant baseline (endogenous) differences in targeted modulators may alter cognitions (e.g., biases toward negative retrieval and/or reduced overall AM retrieval) and potential efficacy of a pharmacological intervention on specific AM retrieval. For example, baseline hair cortisol is positively correlated with PTSD symptoms and sleep disturbances in those with subsequent trauma exposure (Sopp et al., 2021).

Depending on the drug class category investigated, exploring the role of neuromodulator systems in specific AM retrieval may also benefit from the concurrent assessment of cue-response latency and physiological arousal. Such measures could be used to help model the potential influence of general cognitive performance and to assess corroborating physiological effects of a pharmacological manipulation alongside AMT performance. Phenomenological experience during retrieval (e.g., subjective happiness vs. distress) should also be considered. For example, the subjective severity of traumatic events (peritraumatic stress) and distress during retrieval of an associated negative AM can have a significant impact on the development and course of PTSD (Vance et al., 2018) and subjectively positive AMs may be protective against symptom development (e.g., Hamlat et al., 2015).

### Clinical implications

There are four clinical implications of the current findings that warrant further investigation in larger and/or more diverse samples. First, low dose hydrocortisone may be used to model overgeneral AM retrieval deficits seen in psychopathology in healthy samples. Such pharmacological approaches to “symptom provocation” are essential for furthering our understanding of the contribution of specific neuropsychological phenotypes to psychopathology. They also have advantages over behavioral methods, which mimic symptoms for relatively brief periods. Secondly, low dose hydrocortisone may also be used to improve AM retrieval deficits in psychological disorders characterized by overgeneral AM memory. Third, a more comprehensive analysis of divergence in tonic cortisol concentrations between healthy and clinical samples, and interaction between baseline levels and drug effects could improve treatment tailoring. Finally, that drugs that target oxytocin and glutamatergic systems may enhance AM specificity in healthy samples.

### Conclusions

Dysfunctional (i.e., overgeneral) AM retrieval is a transdiagnostic symptom in psychopathology and therefore may be an ideal target for the development of novel pharmacotherapies. Psychotherapies and trauma-focused CBT are the current most effective treatments for MDD and PTSD, respectively (McPherson and Hengartner, 2019; Mavranezouli et al., 2020). The beneficial effects of these treatments has been suggested to be rooted in AM processing, in either the formation and strengthening of competing adaptive AM representations (i.e., self-related knowledge and memories for personal experiences) (Brewin, 2006), or through a “rewriting” process that adaptively alters and/or updates maladaptive AM representations *via* memory reconsolidation (Lane et al., 2015). The development of more-effective psychological interventions specifically targeting AM retrieval (e.g., Memory Specificity Training; Raes et al., 2009a) is likely to benefit from *pharmacological* approaches, as these represent a novel pathway to strengthening our mechanistic understanding of AM specificity and potential catalysts for psychological approaches.

## Data availability statement

The original contributions presented in the study are included in the article/Supplementary material, further inquiries can be directed to the corresponding author.

## Author contributions

EC and SK designed and conducted the systematic review and wrote the article for publication. EC and GP extracted data and assessed risk of bias. SK supervised the study. All authors contributed to the article and approved the submitted version.

## Funding

This research was conducted at UCL and funded by the Sir Bobby Charlton Foundation (Registered Charity No. 114091).

## Conflict of interest

The authors declare that the research was conducted in the absence of any commercial or financial relationships that could be construed as a potential conflict of interest.

## Publisher's note

All claims expressed in this article are solely those of the authors and do not necessarily represent those of their affiliated organizations, or those of the publisher, the editors and the reviewers. Any product that may be evaluated in this article, or claim that may be made by its manufacturer, is not guaranteed or endorsed by the publisher.
