# Supplementary figures and images for: A systematic review of the pharmacological modulation of autobiographical memory specificity
Source: Front Psychol. 2022 Nov 14;13:1045217. doi: 10.3389/fpsyg.2022.1045217 (PMC9703074; doi:10.3389/fpsyg.2022.1045217)

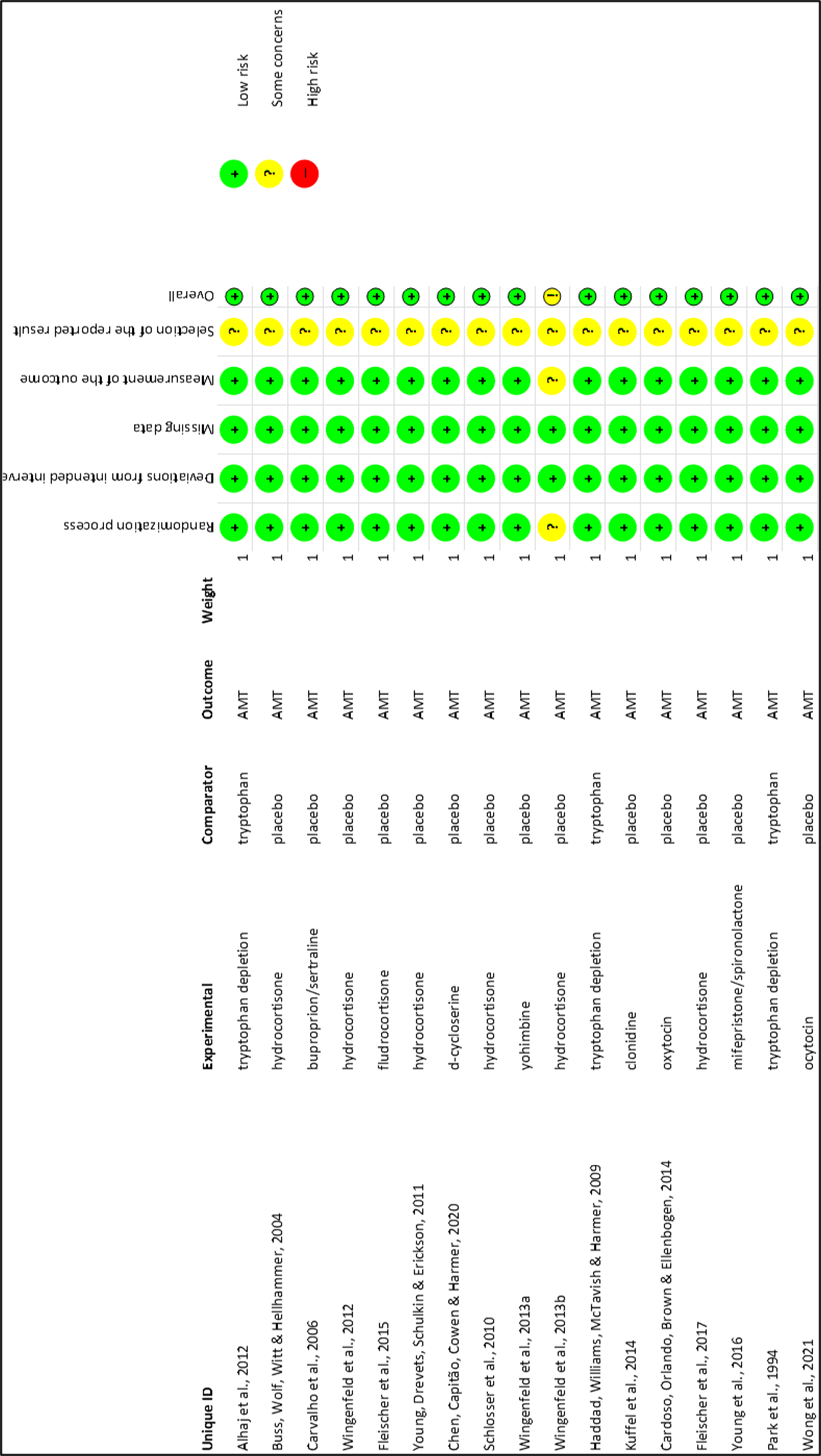

Supplement: Supplementary file 1 [file Image_1.PNG]
